# Application of the wildland fire emissions inventory system to estimate fire emissions on forest lands of the United States

**DOI:** 10.1186/s13021-024-00274-0

**Published:** 2024-08-14

**Authors:** James E. Smith, Michael Billmire, Nancy H.F. French, Grant M. Domke

**Affiliations:** 1 USDA Forest Service, Northern Research Station, 271 Mast Road, Durham, NH 03824 USA; 2 Michigan Technological University, Michigan Tech Research Institute, 3600 Green Ct., Suite 100, Ann Arbor, MI 48105 USA; 3 USDA Forest Service, Northern Research Station, 1992 Folwell Avenue, St. Paul, MN 55108 USA

**Keywords:** Wildland fire, Forest fire emissions estimates, Inventory, United States forest carbon

## Abstract

**Background:**

Forests are significant terrestrial biomes for carbon storage, and annual carbon accumulation of forest biomass contributes offsets affecting net greenhouse gases in the atmosphere. The immediate loss of stored carbon through fire on forest lands reduces the annual offsets provided by forests. As such, the United States reporting includes annual estimates of direct fire emissions in conjunction with the overall forest stock and change estimates as a part of national greenhouse gas inventories within the United Nations Framework Convention on Climate Change. Forest fire emissions reported for the United States, such as the 129 Tg CO_2_ reported for 2022, are based on the Wildland Fire Emissions Inventory System (WFEIS). Current WFEIS estimates are included in the Inventory of U.S. Greenhouse Gas Emissions and Sinks: 1990–2022 published in 2024 by the United States Environmental Protection Agency. Here, we describe WFEIS the fire emissions inventory system we used to address current information needs, and an analysis to confirm compatibility of carbon mass between estimated forest fire emissions and carbon in forest stocks.

**Results:**

The summaries of emissions from forests are consistent with previous reports that show rates and interannual variability in emissions and forest land area burned are generally greater in recent years relative to the 1990s. Both emissions and interannual variability are greater in the western United States. The years with the highest CO_2_ emissions from forest fires on the 48 conterminous states plus Alaska were 2004, 2005, and 2015. In some years, Alaska emissions exceed those of the 48 conterminous states, such as in 2022, for example. Comparison of forest fire emission to forest carbon stocks indicate there is unlikely any serious disconnect between inventory and fire emissions estimates.

**Conclusions:**

The WFEIS system is a user-driven approach made available via a web browser. Model results are compatible with the scope and reporting needs of the annual national greenhouse gas inventories.

**Supplementary Information:**

The online version contains supplementary material available at 10.1186/s13021-024-00274-0.

## Background

Forest biomes are recognized as the most significant terrestrial stores of carbon, and the net accumulation of carbon associated with annual forest growth represents a partial offset to the release of greenhouse gases into the atmosphere. At the same time, the annual extent of forest fires results in a loss of stored carbon reducing the forest offset and affecting atmospheric greenhouse gases [1–4]. For a comparison on forest lands of the United States, in 2022 forest fires emitted 129 Tg CO_2_ while the annual net accumulation on forest lands was 694 Tg CO_2_ [5]. Note that for these two separately estimated totals, the net accumulation on forest ecosystems already implicitly includes effects of fire. These estimates are from comprehensive annual reporting of greenhouse gas inventories for the United States through the Inventory of U.S. Greenhouse Gas Emissions and Sinks: 1990–2022 [5], which includes the contributions of forest lands, as a part of the United States’ commitment to annual inventory reporting within the United Nations Framework Convention on Climate Change [6]. This annual reporting, which includes forest greenhouse gas inventories, is also known as the National Greenhouse Gas Inventory Report, or NIR. The current NIR, which includes the forest fire emissions described here (i.e., U.S. EPA 2024 [5]) will be referenced as NIR below. The NIR includes trends in effects of forest fires over past years; that is, 33 years of estimated net annual CO_2_ emissions (1990–2022). In this publication, we describe the current approach and identify information needs in developing the fire estimates, which is principally through application of the Wildland Fire Emissions Inventory System (WFEIS, [7]).

Fires on forest lands have impacts beyond their effect on the balance of greenhouse gases between the land and atmosphere. For example, fires affect the health and safety of people living on or near forest lands [8–10]; impact those who directly rely on forest ecosystems or the forest-related economy [11, 12]; as well as effect change in forest ecosystems such as soil properties, component species, or stand structure, for example [13–16]. Related to the diversity in forest fire impacts, simulations developed to characterize forest or wildland fires can have diverse intended applications at a range of scales, often at regional scales. For example, assessments address fire risk [17]; generation of smoke or particulate matter [18–20]; spread or behavior of active fires [21]; economic or ecosystem services effects [12]; or effects on forest ecosystems [22–24]. Many such models include components that estimate emissions. However, for purposes of greenhouse gas reporting [5, 6], estimated emissions need to conform to whole-country forest land bounds and be applicable to current and past years as included in the NIR.

Multi-year assessments of wildland or forest fire emissions at a global scale [2, 25–27] can be applied as coarse-grained country level emissions inventories. However, records associated with specific forest land and subsequent use of these data for simulations of fuel, biomass burned, and allocation to emissions can produce estimates more characteristic of United States forests. Published assessments of emissions from fires on forest land for the United States that are then repeated for multiple specific years include several studies [7, 28–32]. In addition, regional assessments or evaluations of alternate approaches have nation-wide potential [17, 33, 34]. While approaches to simulations and model-specific datasets vary, estimates are generally based on a few essential steps [4, 35, 36], and these include defining: (1) date, location, and size of forest burned (‘activity’); (2) site conditions and the state of available fuel; (3) burn characteristics of the fire and biomass consumed; and (4) allocation to emission of CO_2_ and other compounds emitted with fires.

Here, we provide a brief overview of WFEIS and describe its application to estimate fire emissions on forest land of the United States. Implementation of this approach for reporting fire emissions represents improved methodology and ongoing efforts toward more country- and fire-specific estimates [35]. Three aspects of the process are addressed. First, we describe our application of the WFEIS-modeled emissions and the available input data sources that delineate past wildland fires. We compare differences among data and the subsequent influence on estimates. Second, we summarize some attributes of fire data that potentially limit accounting for all fires; we specifically address smaller fires and prescribed fires. Third, we provide a first analysis of consistency between the WFEIS-based fire emissions and the inventory-plot based estimates of forest carbon stocks within these fires, which are largely independently obtained quantities.

## Methods

### Wildland burned areas data

**Fig. 1:**
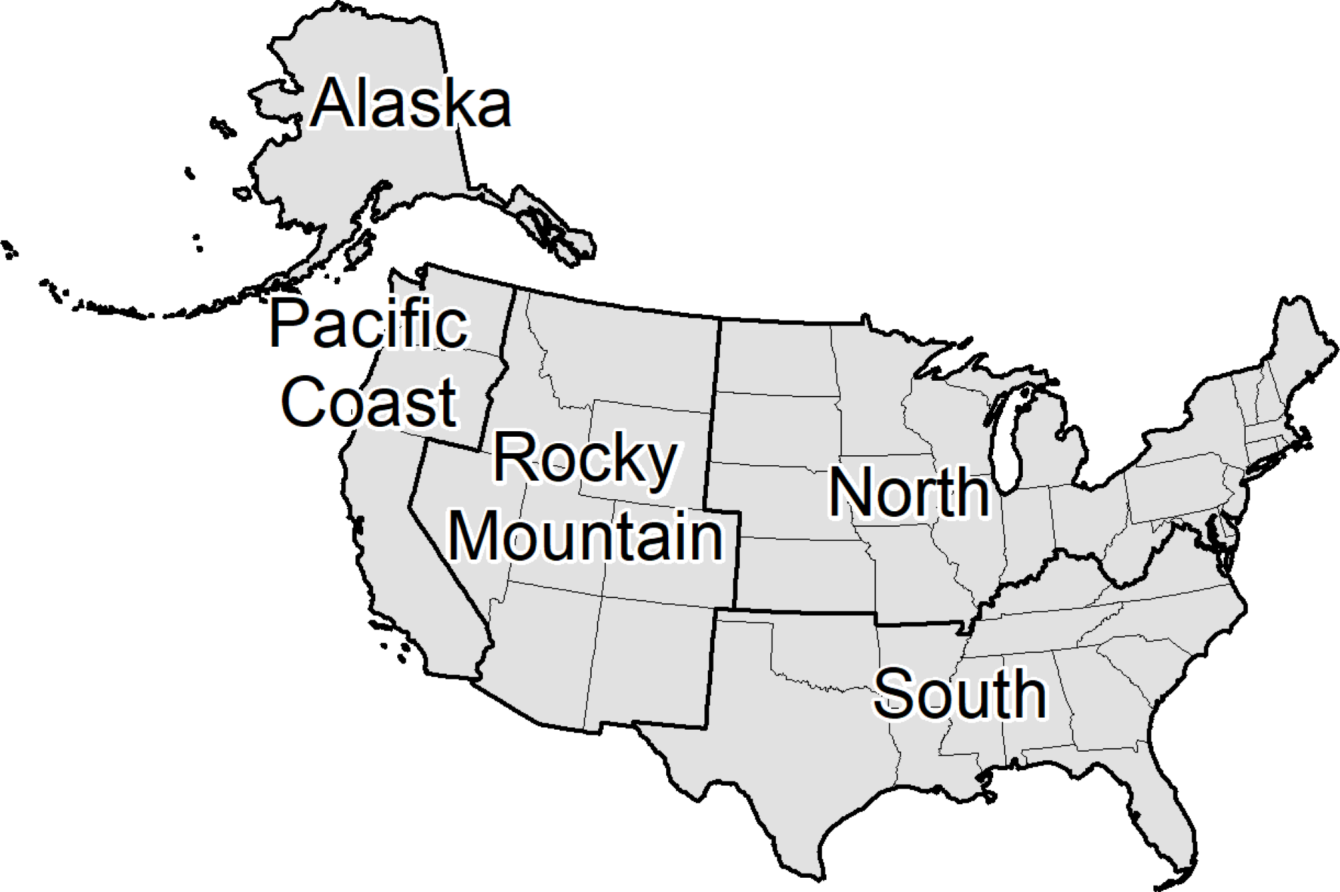
Geographic regions and component states used for summarizing data

Spatial definitions of wildland fire burned areas are the primary data for compiling past forest fire emissions. We include three sources of datasets defining individual burn perimeters and dates of fires on United States forest lands within the 48 conterminous states (CONUS) and Alaska (Fig. 1). The current NIR [5] includes forest fire emissions for 1990–2022, and years of the summaries provided here vary, depending on data availability.

The Monitoring Trends in Burn Severity (MTBS, [37]) burn perimeter data are based on burn occurrence records, Landsat reflectance images, and comparison of pre- and post-fire burn indexes [37, 38]. These data are developed for national level analyses, and the data include the years 1984 through 2021, with 1990–2021 used here for most states. The minimum area per burn included in the data records are approximately 400 or 200 ha (1000 or 500 acres), for western or eastern fires, respectively. For MTBS, western states include those between North Dakota and Texas and all others to the west including Alaska.

The second burn source in use here is the Moderate Resolution Imaging Spectroradiometer (MODIS) burned area mapping product (MODIS MCD64A1, [39]). MODIS burned areas are based on daily surface reflectance and detection of rapid changes in reflectance associated with recent fires, which provides date of burning, spatial extent of fires, and distinction of recent versus past season fires (Giglio et al. 2018). Years included for this analysis include 2001 through 2022.

A third burn source is the Wildland Fire Interagency Geospatial Service (WFIGS) Interagency Fire Perimeters obtained from the National Interagency Fire Center website [40]. Burn perimeters are mapped through a variety of approaches; see National Interagency Fire Center [40] for additional information. The years 2021 and 2022 appeared to be complete and are included here.

The MTBS data were downloaded directly from the MTBS site in August 2023 (the August 2023 update, [41]). The MODIS burned areas were downloaded through the WFEIS calculator [43] in June and July 2023; these maps are obtained for WFEIS from the Land Processes Distributed Active Archive Center [42]. The WFIGS records were downloaded from National Interagency Fire Center [40] in June 2023. See Table S1 for an example of differences among the perimeter sources, particularly with respect to burn size and number of records. Ostensibly, all layers include all wildland fires with the exception of the smaller fires below the MTBS minimum size threshold; those smaller fires are potentially included in MODIS and WFIGS.

### The wildland fire emissions inventory system

Simulations of emission reported for past forest fires are from the WFEIS system of models, which were developed to estimate fire emissions based on fuel consumption in historical fires of the United States and Canada and has been extended to additional countries. Emissions estimates are made available for queries from a web browser [43]. The system provides multiple combinations of inputs to identify and delineate fires and site characteristics. The process is intended to provide consistent estimates at scales from individual forest or county to regional or national summaries. Initial geospatial specification of fire location, date, and size are available for selection from several data sources, or even user-defined fire perimeter layers. Within the burned area, fuel source information is used to characterize the burn site [7], with two alternate sources used for the U.S. estimates. The Fuel Characteristic Classification System (FCCS) layer provides mapped LANDFIRE Existing Vegetation Types [44, 45]. The North American Wildland Fuels Database (NAWFD) models variability in mapped fuel type by aggregating fuel loading data from many sites and data sources, which provide means to quantify uncertainty by type and location [46]. Fuel is further characterized according to location and date of burn by modelling fuel moisture [25, 28]. Modeled moisture inputs are from two daily gridded weather models: gridMET [47], which provides daily 4-km 1000-hr dead fuel moisture data for CONUS, and the Global Fire WEather Database [48], which provides daily 0.5 degree resolution. Fuel consumption and emissions are predicted by the simulation Consume [49]. Emission factors internal to Consume allocate emissions to CO_2_ and other non-CO_2_ greenhouse gasses.

All WFEIS estimates were obtained through queries of the WFEIS Calculator [43]; the scripted queries used urllib, a Python library for http requests. We primarily collected whole-state annual estimates for combinations of burn source (MTBS, MODIS, WFIGS) by fuel source (FCCS, NAWFD); availability varied by source by year. Note that the MTBS-based estimates for Alaska 2021 were not available for the NIR and are also omitted from these summaries. Additional smaller area-of-interest queries are defined by the WFEIS calculator such as by county or by boundaries of specific National Forests. Additionally, estimates per burn perimeter are available and these were queried for the MTBS and WFIGS sources. Results included as many as six separate whole-state emissions estimates for some state by year combinations and as few as two for others (e.g., see Tables S2 and S3.

Our focus is specifically forest fire emissions [5], yet WFEIS simulations include wildland fires on all land types. Therefore, forest fuel types were extracted for analysis based on the Landfire existing vegetation type [45], which is integrated into WFEIS. Results were collected as tabular summaries according to fuel classification. The subset of fuel classes within each of the fuel sources that are identified as ‘forest’ were used to identify forest burned area and forest-specific emissions for the complete wildland burned area. This approach provides a nonspatial aggregate of forest fire area and emissions within each query; that is, within state-level or individual burn perimeters (depending on queries).

### Additional classification of WFEIS-returned emissions

Spatially accounting for large-fire emissions where perimeters extend over more than one domain of interest (e.g., state lines or National Forest boundaries) requires spatial allocation to these alternate domains. In these cases, emissions are allocated according to relative proportion of forest cover in each domain within the large burned area according to forest cover of the Multi-Resolution Land Characteristics National Land Cover Database [50, 51]. Forest cover classes define areas that generally aligned with the per state forest area of Oswalt et al. [52], yet their sole purpose here is disaggregation of individual fires.

In temperate forest ecosystems, carbon emitted through fire is closely related to standing tree carbon, and the two quantities defined in the NIR are largely independently obtained. The intersection of forest inventory plots with MTBS forest fire perimeters provide the means to roughly compare relative magnitude of the two quantities. To assess mass of carbon emitted relative to pre-fire tree carbon stocks, aboveground live tree carbon stocks (Mg C ha^− 1^) were obtained from permanent forest inventory plots (Forest Inventory and Analysis Database, [53]). We identified MTBS burn perimeters from years 2011–2020 that included both (1) forest fire within the perimeter and (2) spatially intersected a permanent forest inventory plot that was measured (plot visit) 5 to 10 years prior to the fire.

This report addresses only area of forest fire and CO_2_ emissions on that area; the corresponding non-CO_2_ emission details are available from other NIR related sources [5, 54, 55]. WFEIS results explicitly include the necessary set of non-CO_2_ emissions for the NIR – CH_4_, CO, and NO_x_ [5]. The WFEIS calculator was queried in May, June, and August 2022, with some repeats of queries to assure that the MTBS-based predictions included fires through 2021.

## Results

### WFEIS fire emissions, multiple estimates

**Fig. 2:**
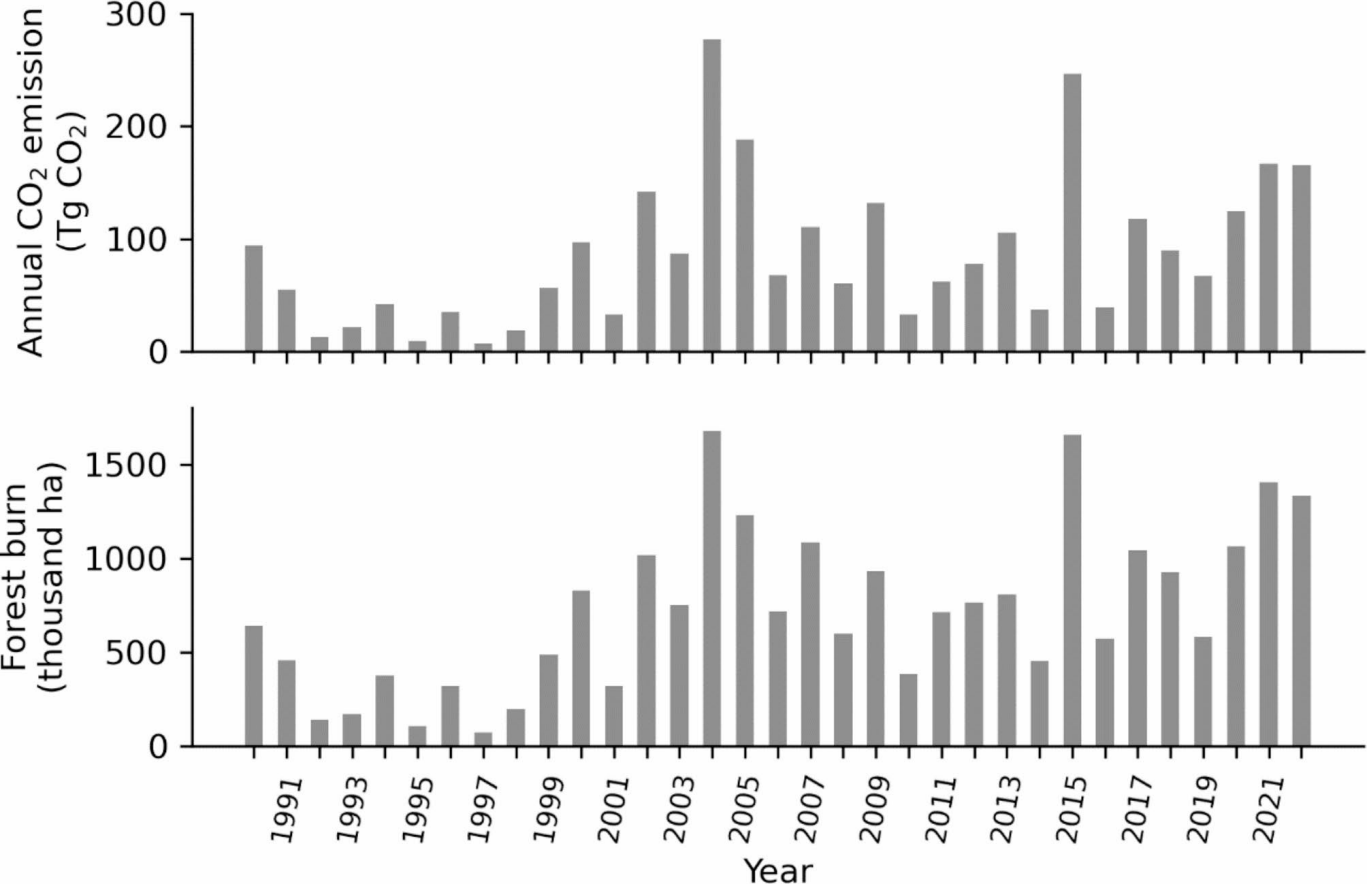
Annual CO_2_ emissions and area of forest fire from WFEIS modeling for CONUS plus Alaska, 1990–2022

Annual CO_2_ emissions from forest fires and annual area of forest burned as predicted by the WFEIS system for forest land from 1990 to 2022 show high year to year variability (Fig. 2). The years with the highest CO_2_ emissions from fires on these forests were 2004, 2005, and 2015. The 1990s clearly have lower fire emissions and area burned relative to the 20 years since.

**Fig. 3:**
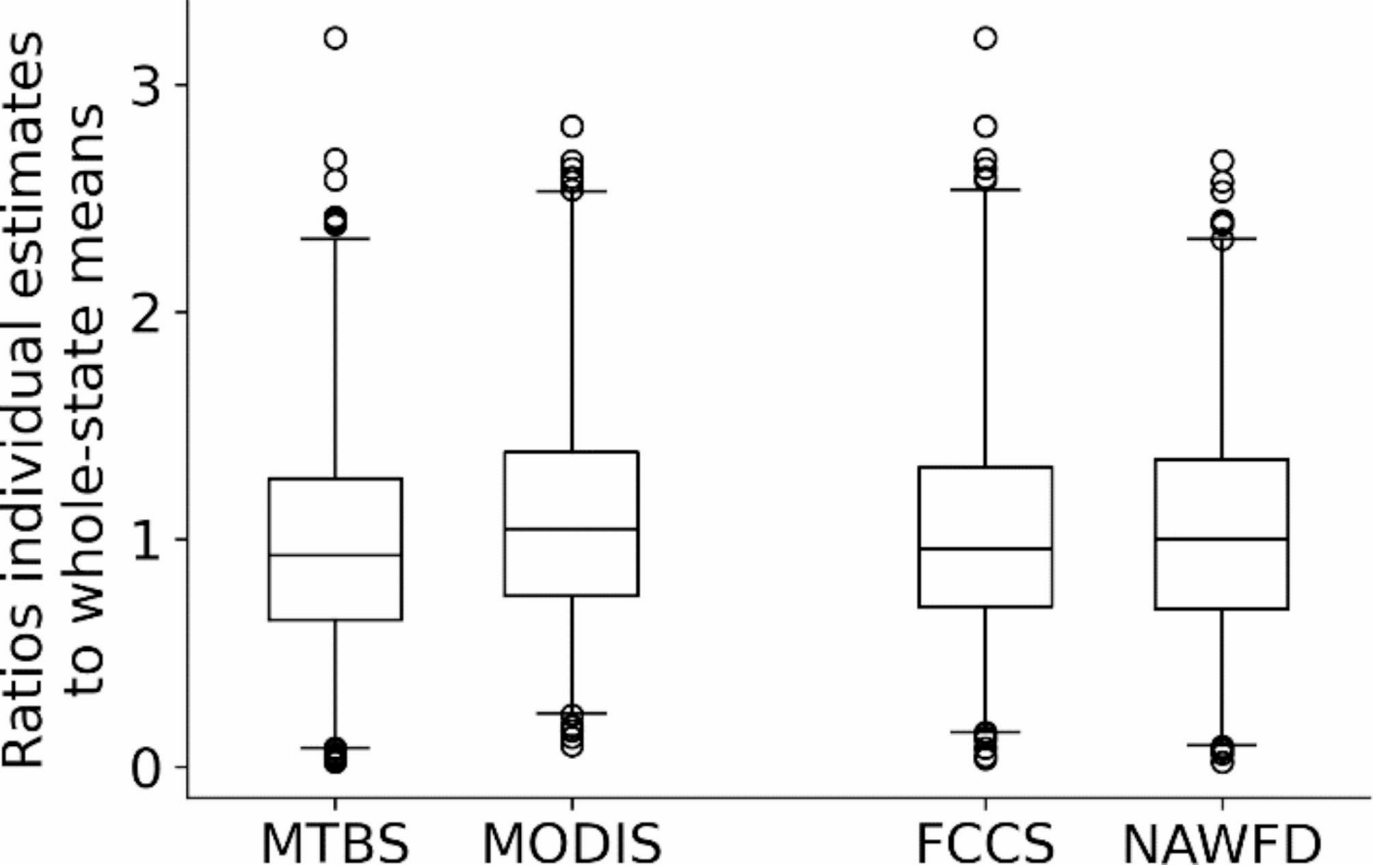
State level ratios of emissions based on combinations of burn source (MTBS or MODIS) or fuel layer (FCCS or NAWFD) to mean of all inputs. Ratios accumulated for all state level forest fire summaries, 2011–2020. Whiskers on plots represent the 1st and 99th percentiles of the ratios

Summarized annual emissions and forest areas (e.g., Fig. 2) are based on means of the state-level annual estimates from WFEIS; two to six separate estimates are available for each year by state depending on availability of burn source data. For example, over the interval from 2001 to 2020, each state level estimate was the mean of the two-by-two combinations of burn source (MTBS, MODIS) and fuel definition (FCCS, NAWFD). The relative dispersion of each of these data sources around the resulting estimate per state (i.e., ratios of the multiple source-based estimates to state means) are shown in Fig. 3, where each box plot is based on two ratios per state and accumulated over 2011 through 2020. The purpose of these plots is to illustrate that while different combinations of model inputs produce differences in emissions, there are not consistent large differences, such as indicated by alignment of the interquartile ranges of Fig. 3. Over the 10 years, average emissions from fires defined by MODIS were slightly greater than those defined by MTBS. Similarly, emissions from NAWFD fuels definitions were slightly greater than the FCCS-based emissions. The burn source layers (MTBS, MODIS, and WFIGS) and the fuel layers (FCCS and NAWFD) can affect annual totals per state. These varying influences are apparent in regionwide annual summaries according to the separate combinations of model inputs to WFEIS (see Tables S2 and S3, which also indicate availability of each burn source according to the cells populated). Overall, for most state and regional annual totals, no single burn or fuel source is associated with consistently greater estimates, but there are some exceptions such as the South where all regional forest areas burned are greater from the FCCS-based estimates. Emissions in western regions are consistently influenced by fuel source, with greater emissions associated with FCCS in Alaska but with NAWFD in Pacific Coast and Rocky Mountain.

The burn records do not delineate forest fire within their respective wildland fire burn perimeters. Because the MTBS and MODIS layers are the primary burn sources, we summarized relative proportions of forest in each as well as where spatially coincident. The land cover images [50, 51] were used to partition perimeters to forest and so compare pixels as coincident or unique to one source or the other. A 10-year average of annual burn perimeters on forest land indicates that the MTBS burns capture a greater area of forest relative to MODIS (Table 1). There are clear regional differences in forest burned between the two sources. The majority of burned area in the West is detected jointly by both sources (MTBS and MODIS), yet a considerable proportion of burned area that is forest is not jointly located by both sets. In general, MTBS identifies a greater amount of annual average forest burn area not detected by MODIS as compared with the burned areas unique to MODIS. Detection in the East showed a different pattern (yet same for North and South) where a majority of forest burned areas are unique to one or the other of the sources and those areas were similar.

**Table 1:** Forest area common to both MTBS and MODIS burn perimeters and forest unique to each

Region	Forest area common to both burn sources	Forest area unique to MTBS	Forest area unique to MODIS
		thousand ha	
Alaska	140 (3, 637)	121 (15, 506)	25 (1, 137)
Pacific Coast	332 (16, 1326)	82 (9,209)	21 (6, 36)
Rocky Mountain	304 (56, 712)	134 (75, 232)	53 (28, 111)
North	5 (1, 25)	8 (4, 12)	9 (3, 18)
South	82 (35, 248)	167 (70, 274)	163 (96, 245)

Mean annual area of NLCD forest cover within MTBS or MODIS burn perimeters, 2011–2020. Values in parentheses represent annual minimum and maximum areas from the 10-year interval

### Possible contributions of smaller fires

In order to quantify the potential role of fires below the MTBS minimum size thresholds we summarize two datasets that include small burned areas. Fire records from 2001 to 2020 of the Spatial Wildfire Occurrence Data [57, 58]. The percentages of these burned areas and count of fires (Table 2) that are under the regional MTBS thresholds suggests that only approximately 1% of fires in the 48 conterminous states are included in MTBS records because of the very large number of small fires. Percentage of burned area indicates that most of the area burned are over the threshold and included in the MTBS data; this is particularly true in the West but less so in the East. Note that Table 2 is based on wildland fires and not the subset of forest fires used in this study. A summary of the below-MTBS-threshold forest fires based on two years of the WFIGS records (Table 3) indicates proportional and relatively small contributions of these fires in terms of burned area and emissions.

**Table 2:** Proportion of wildland burned areas below the MTBS minimum size thresholds, from spatial wildfire occurrence data records

Region	Percentage of burned area	Percentage of fires (count)
Alaska	1.0	89.7
Pacific Coast	5.2	99.2
Rocky Mountain	8.4	98.7
North	49.7	99.8
South	33.3	99.5

Mean annual percentages from the Spatial Wildfire Occurrence Data records [57] within regions where the burn area is below the threshold for MTBS reporting summarized for fires 2001 through 2020. Note that this summary is based on all wildland fire records and not limited to forest fires as are most other summaries included here

**Table 3:** Forest fires in burned areas below MTBS minimum size thresholds based WFIGS 2021 and 2022

Region	Forest fire	CO_2_ emitted	Number of fires
	thousand ha	Tg CO_2_	
Alaska	29.6 (6.7)	4.6 (6.8)	102 (62.3)
Pacific Coast	51.9 (7.3)	5.9 (5.8)	334 (79.9)
Rocky Mountain	54.3 (14.2)	5.0 (14.7)	628 (79.8)
North	8.6 (45.8)	0.7 (32.6)	577 (95.6)
South	30.2 (46.6)	1.7 (43.7)	803 (86.9)

Mean annual sum (percentage of all forest fires represented in the column)

### Contribution of prescribed fires

Identifying location and origin of emissions from forest fire can be useful for some sub-national reporting (i.e., managed versus unmanged land and anthropogenic versus natural origin) [6]. Records in the MTBS data identified as ‘prescribed’ are used to estimate annual area of prescribed forest fires (Table 4). Similar summaries for the additional classification of ‘unknown’ origin are also included in Table 4. In general, area of prescribed forest fires increased between the two time-intervals, and area and overall proportion of such fires is greater in the East, particularly in the South. The unknown origin classification of forest fires proportionally decreased between the two time-intervals. In 2020, 182 thousand ha, or 8% of the MTBS forest fires are identified as prescribed. A summary of annual prescribed forest fire extracted from the 2021 and 2022 WFIGS burn perimeter records (Table 5) shows essentially similar regional results, with slightly different areas and proportions. The WFIGS records also make it possible to summarize prescribed forest fires below the minimum size threshold for inclusion in the MTBS records. The majority of the prescribed forest fires (in WFIGS, Table 5) were below the MTBS threshold in three of the CONUS regions – Pacific Coast, Rocky Mountain, and North. This proportion is about 20% in the South, which is the region with the greatest total and total below threshold areas of prescribed.

**Table 4:** Prescribed and unknown-origin annual forest fires classifications; forest burned areas based on MTBS.

Region	1990–2005	1990–2005	2006–2021	2006–2021
	Forest burned, prescribed	Forest burned, unknown origin	Forest burned, prescribed	Forest burned, unknown origin
		thousand ha		
Alaska	0 (0)	12.9 (5.2)	2.0 (1.2)	0 (0)
Pacific Coast	1.7 (3.0)	0.1 (0.2)	3.0 (2.0)	1.0 (0.6)
Rocky Mountain	4.5 (4.0)	3.9 (3.4)	9.1 (4.7)	6.5 (3.4)
North	1.2 (4.8)	5.1 (21.0)	7.1 (45.8)	1.0 (6.7)
South	5.2 (6.7)	37.0 (47.7)	132.4 (60.4)	22.6 (10.3)

Mean annual sum (percentage of all forest fires represented in the column)

**Table 5:** Prescribed annual forest fires classifications; area based on WFIGS, 2021 and 2022

Region	Forest burned	Forest burned -subset below MTBS minimum
	thousand ha	
Alaska	4.2 (1.0)	1.2 (28.2)
Pacific Coast	0.7 (0.1)	0.4 (54.5)
Rocky Mountain	0.9 (0.2)	0.9 (100)
North	5.9 (31.4)	3.3 (56.1)
South	25.4 (39.2)	5.9 (23.4)

Mean annual sum (percentage of all forest fires represented in the column)

### Fire emissions relative to forest inventory carbon

**Fig. 4:**
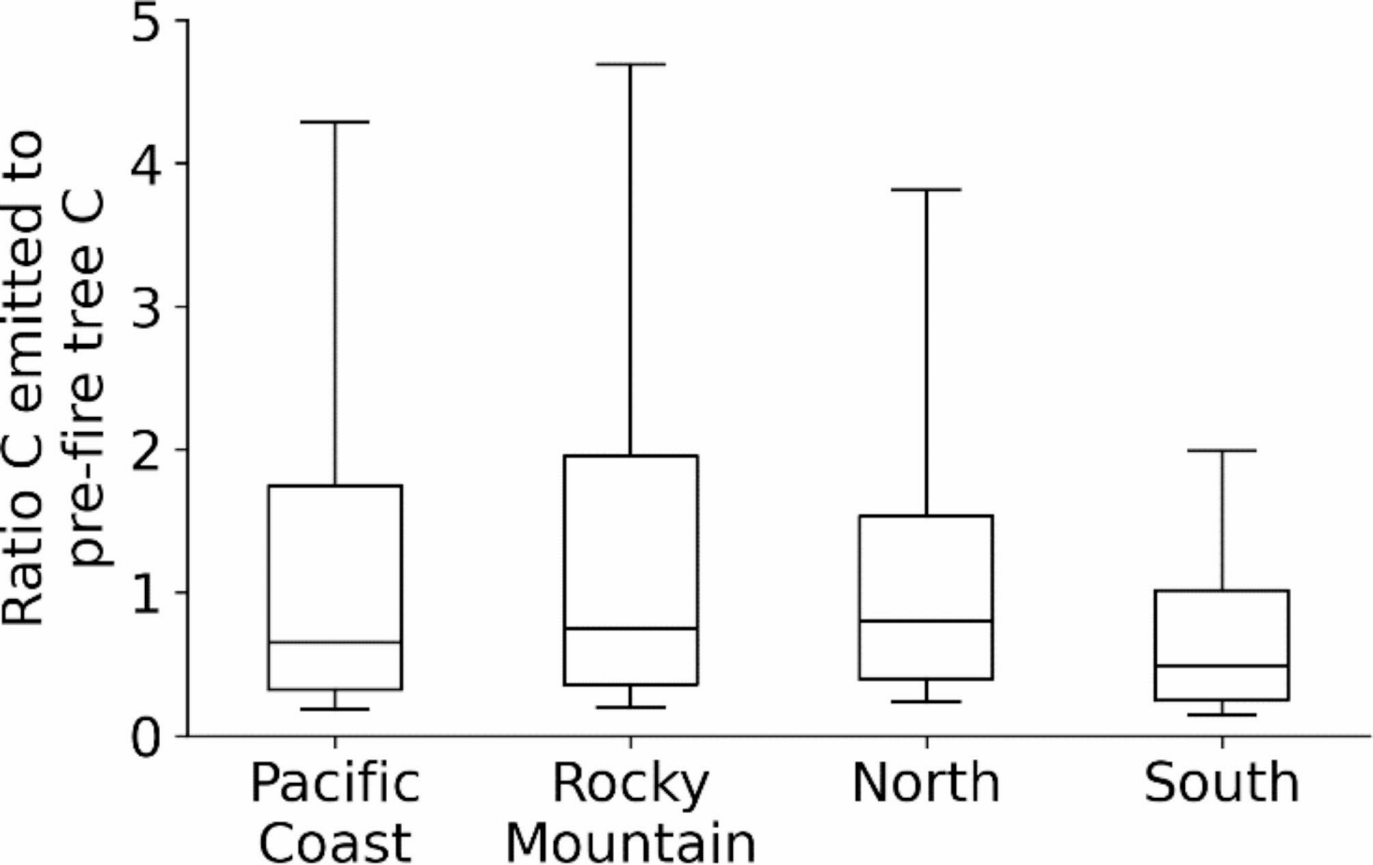
Ratios of mean carbon emitted (Mg C ha^− 1^) from MTBS-defined forest fires to pre-fire aboveground tree carbon (Mg C ha^− 1^) on inventory plots over those fires. Whiskers on plots represent the 10th and 90th percentiles of the ratios

The data and models used to estimate forest fire emissions (via WFEIS) are not directly linked to the data or models used to estimate forest carbon stocks (via forest inventory, [53]). To determine if the two separately determined quantities are broadly comparable (e.g., same magnitude) we provide an informal analysis based on the intersection of the data (i.e., temporally and spatially, Fig. 4). The ratios of emitted carbon to tree carbon provide a single point intersection between two spatially variable features. That is, actual values for both the numerator and denominator are spatially variable across burned areas. It is beyond the scope of this summary and currently available data to refine these ratios, but the values provide a distribution – many small and few large – that plausibly suggest general equivalence. Regional medians are greater than 0.5 and less than 1.0 (Fig. 4).

## Discussion

### WFEIS and forest fires

These applications of the WFEIS calculator and the resulting forest fire and emissions summaries follow the basic greenhouse gas reporting paradigm of the Intergovernmental Panel on Climate Change (IPCC) guidance for reporting [35], which is essentially a product of two broadly defined factors: emissions = activity data × specific emissions. The first such use of WFEIS to simulate these factors was with U.S. EPA in 2022 [59], and this approach substantially refined the U.S.-specific representation of fuel and combustion relative to fire emissions estimates of previous NIRs (i.e., pre-2022).

The forest fire emissions estimates provided here and those in the 2024 NIR are compiled on the same set of WFEIS queries and calculations. The purpose here is to address the WFEIS results as they apply for national and sub-national reporting, and not to repeat the previously published fire emissions. However, for perspective the NIR totals include CONUS, Alaska, Hawaii, and U.S. territories, which was 129.2 Tg CO_2_ emitted for 2022 [5]. The summary proved here does not include Hawaii or Guam (< 0.002 Tg CO_2_ and the only additional forest fires reported for 2022), and it includes 93.7 Tg CO_2_ emitted from all Alaska forest lands in 2022 rather than the 57.4 Tg CO_2_ on managed Alaska forestlands, as in the NIR. The resulting total for CONUS and Alaska in 2022 is 165.5 Tg CO_2_ (Fig. 2).

The WFEIS system has been continuously updated since it became available. In recent years these updates include the current FCCS fuels layer provided by the USGS Landfire data and addition of the NAWFD model as well as changes in fire source data available through links in the calculator [43]. WFEIS is well suited for the scope of the reporting needs of the NIR [5]. Note that queries for Hawaii fires are available and will be incorporated into future NIRs.

Current WFEIS estimates are broadly consistent with previous emissions observations, and they include high interannual variability and a trend toward recent greater average annual area burned relative to the 1990s. Burned forest area is apparently smaller throughout the 1990s as compared with the more recent twenty years (Fig. 2). This may be related to an increase in number or extent of fires in the recent years, but Hawbaker et al. [34] also suggest that Landsat burned area products provide conservative estimates prior to 2000 when all detection was by the Thematic Mapper sensor. The estimates for direct carbon emissions of forest fires (French et al. 2011, supplemental Table 1) were generally similar but slightly less than the current estimates (e.g., Fig. 2); differences are likely attributed to the method of selecting the forest subset and to changes in MTBS early-2000s perimeter records versus current data. That is, between MTBS available in 2011 versus the 2022 compilation [38]. Other similar scope estimates of annual emissions from fires over CONUS, for example, identify forest lands in order to estimate fuel and emissions or as a part of spatial summaries but do not separately tabulate forest fire emissions [29, 30, 32]. Larkin et al. [56] and Urbanski et al. [31] include wildland fire emissions, but without CO_2_ emissions on forest land, they are not parallel assessments.

Forest fire emissions reported here represent the effect of fires on forest carbon stocks within each year (i.e., net of accumulation and fire emission). The fire-related emissions estimates from WFEIS and reported in the NIR [5] are modeled as emissions released to the atmosphere during the fire (i.e., immediate). As such, they do not represent total ecosystem carbon loss resulting from the fire disturbance; that total requires a multi-year time frame to account for all the delayed effects [60]. The longer-term, or delayed, emissions are primarily from years of decomposition of the post-fire burned and unburned dead woody material, standing or on the ground. Long-term total effects of fire – immediate and delayed – are captured in remeasurement of inventory plots, which continue many years after a fire. From this, stocks and annual increments of the forest greenhouse gas inventory in the NIR [5] include fire effects on forest ecosystem carbon pools, primarily biomass and dead wood for most temperate forests.

The combinations of the basic WFEIS inputs – burn and fuel layers – can produce two to six separate burned area and emissions estimates, depending on data availability (see Tables S2 and S3). Each estimate is a single-value, or deterministic, prediction, and the current approach is to set the mean of available estimates as the summary estimate for each state. These are then summed to the national total. A clearer understanding of how the input layers are related would be useful prior to developing alternate approaches to pooling or defining uncertainty. While the only clearly defined difference in qualifying a fire to be included in the respective layers is the size limits of the MTBS, there is currently no consistent means of identifying specific fires common, or alternately, unique among the sets. At the regional level, we saw that greater variability in projected CO_2_ emissions is associated with the fuel layers than with the burn sources, and relative differences are greater in the western regions. In general, fuel type models used to simulate fire emissions have been identified as having a high influence on modeled estimates [61–63]. Our current approach to using the two fuel sources (FCCS and NAWFD) was effectively to take one prediction based on each and pool the resulting predicted emissions. Any future changes to how the multiple burned area and fuels layers are used for compiling the U.S. emissions estimates partly depends on resolving the similarities and differences. The potential for quantifying uncertainty or incorporating uncertainty analysis into estimates has been reviewed by French et al. [64] and Prichard et al. [46].

### Burned areas

High variability among alternate sources delineating fire [3, 34, 39, 65] can occur partly due to different methodologies (variously defined by ground based, aerial, or satellite mapping methods), and these differences are carried through estimated emissions. Wildland fire records for the United States are compiled from multiple sources including agencies responsible for land management or agencies responding to wildfires, and these data are frequently available in geospatial datasets. In addition to the three spatial layers in use here, the National Interagency Fire Center [66] collects and compiles spatial data from fire incidents and provides annual summary statistics on wildland fires (currently for 1983–2022). Similar and related compilations include the Combined Wildland Fire Dataset [67], currently for 1835–2020), and the Spatial Wildfire Occurrence Data for the United States ( [57], currently for 1992–2020). As with the MTBS and WFIGS, these summaries are compiled from multiple sources, and therefore, there is considerable overlap among them in underlying data and defined burn records.

Overall total burned area per year from each – MTBS and MODIS – represent partly disjoint spatial datasets, and the extent of spatial agreement varies by year and region despite ostensibly seeing the same fires. A large proportion of these areas are spatially coincident burns from both sources (Table 1). Summed annual MODIS-based burn area is greater than that of MTBS for 19 of the 20 years in common for both sources (i.e., 2001–2020). In contrast, the area of MTBS burn perimeters was greater than those of MODIS for all 20 years in Alaska. Just as neither one captures all burns in the other source, there is no reason to assume that the union of the two captures all wildland fires each year. The different sources and mechanisms for delineating burns means that inclusion of a burn can be variable between the two sets for possibly less well-defined or less detectable fires [38, 39, 68]. However, most wildland burns are probably accounted for because of the similarity in burned areas among other such sources of compiled fire data. Relative to the to 4.30 million ha in MTBS and 5.08 million ha in MODIS, other totals for CONUS plus Alaska in 2020 are 4.25 million ha in Short [57], 4.27 million ha in Welty and Jeffries [67], and 4.10 million ha in National Interagency Fire Center [66].

Forest fires within the larger wildland burn perimeters are reported based on the WFEIS fuel layers. However, the spatial overlay of burn perimeters with land cover provides toward disaggregation of fires, and it also provides consistent spatial subsets of MTBS versus MODIS perimeters that represent likely area of forest fire. The allocation according to land cover in Table 1 indicates that despite the many fires identified by both sources, there are clear differences among forest fires located by each. Differences include an East versus West effect with a higher proportion of forest fire common to the two sources in the West, relative to the East. From the same (Table 1) analysis, in 2020 for example the mean proportions of MTBS and MODIS burn perimeters with forest cover are 53 and 38%, respectively. Resolution of how the fuel-class based Consume simulated forest fires are related to potential forest fire within perimeters from landcover images can improve finer scale apportioning of emissions. This applies to allocating fires to different domains of interest or to accounting for unburned islands within perimeters [69, 70]. This is beyond the scope of this analysis but indicate areas of improvement in future assessments.

The summaries of the intersection of MTBS, MODIS, and forest land cover when viewed as regional summaries do suggest that there may be burned areas unique to each of the sources. If this is true, a union of the two sets that avoids double counting may be useful for more complete reporting, but this is beyond the scope of this analysis. A first step in resolving fire records into a pooled MTBS-MODIS set is to recast groups of MODIS perimeters into grouped single fire events following Balch et al. [71] for comparison with the MTBS events. Each MODIS perimeter (scene) includes a start date for the burn, which is useful for characterizing a large fire by including temporal as well as spatial similarities. The MTBS records only provide a single start date, which can make it difficult to conclusively link all MODIS perimeters in the proximity of a MTBS perimeter if the MODIS burns extend over many days. Without those added levels of modeling to properly merge and co-locate alternate fire data, the most appropriate use of the MTBS and MODIS sources are as two alternate representations, done in this study. Similarly, any evaluation of the uniquely MODIS perimeters as below MTBS thresholds necessarily depends on first resolving distinct burn events in burn layers.

The relative contributions of Alaska forest fires to totals are slightly greater here as compared to the NIR and Walters et al. [55] because Alaska fires here include all forest land rather than managed lands only [5, 72]. Annual emissions on unmanaged forest lands, which are deducted from NIR totals, varies by year from a low of 29% of the Alaska total in 2017 to a high proportion of 90% in 2001. An average of 64% of Alaska emissions over the 1990–2022 interval were on unmanaged forest lands (data not shown). For reference compare Tables S2 and S3 with the Alaska summary in Walters et al. [55].

### Burn size

A consistent relative difference in numbers and locations of fires among alternate burn source datasets can suggest a significant contribution of possibly smaller fires if unique to a set [34]. However, this is not readily apparent with the burn sources in use here; Table 1 indicates large areas unique to either MTBS or MODIS. While some of the MODIS-only areas are likely under-MTBS-threshold burns, there are potentially many burned areas below the MODIS minimum (i.e., 500 m or 25 ha of a single scene) that are not included in Table 1 as suggested by Table 2. Effects of these smaller fires can collectively contribute substantially to total emissions [73]. The relative roles of smaller fires in the Spatial Wildfire Occurrence Data [57] and WFIGS [40] data suggest that those very many smaller fires can account for a substantial proportion of burned area and emissions, particularly in the East. The actual proportions of small fires in these data are slightly more than indicated here because prior to extracting the below-threshold subsets from the Spatial Wildfire Occurrence Data and WFIGS data, we dropped records with burned area less than 0.2 ha and within 100 m of each other on the same day, which set a consistent lower bound for the two sets.

### Prescribed forest fires

There is an interest in reporting emissions associated with prescribed forest fires. Current forest emissions reporting of the NIR [5] does not separately identify a proportion of forest fire emissions originating from prescribed burning. This is principally because of an apparent wide and unresolved gaps among data sources in the amount of burned area labeled prescribed [32, 41, 74, 75]. The information available within the current system is the incident type identifier field in the MTBS records, which for example, totals 328 thousand ha in 2019. Of this total, we estimate that 234 thousand ha are forest. These areas are considerably lower than other summaries for the same year [32, 74, 75]. A contributing factor in the lower level of prescribed classifications in the MTBS records is the lack of consistent prescribed fire records among states. As a result, state-prescribed fire records are not included in MTBS burn assessments [38]. Inclusion or identification of prescribed burn records is not a part of most of the geospatial summaries currently available. Welty and Jeffries [67] identify 569 thousand ha prescribed burn in 2019 for CONUS plus Alaska, and some of the prescribed records in these data note the difficulty in fully reporting prescribed areas, which may contribute to underrepresenting actual areas. The Inter-Agency Fire Perimeter History spatial layer (available at [65]) includes 31 thousand ha of prescribed fire perimeters in California for 2019 among the larger set of all CONUS plus Alaska perimeters. The tabular annual statistics at National Interagency Fire Center list 2.45 million ha of prescribed fires in 2019 [65]. The 2020 National Prescribed Fire Use Report [74], prepared by the Coalition of Prescribed Fire Councils, lists 4.05 million ha of prescribed burn on forest and range land for the 50 states in 2019. This magnitude of prescribed burn corresponds with summaries for 2011 and 2014 by Larkin et al. [32]. The underlying individual burn information on location, date, and size are not always readily available for the reports of annual totals; these would be useful for first steps in resolving differences among data sets.

Better resolution of prescribed burns on forest land is a priority for future NIR reporting. A practical first step is compiling any form of available records of prescribed burns on forest land that include at least date and location. Additionally, burn size and spatial information would be useful. The records of prescribed forest fires can be compared with various fire datasets (e.g., MTBS, WFIGS, Welty and Jeffries [67], Short [57], or MODIS) to possibly label where records correspond to the prescribed burn list. As prescribed forest fires are identified and characterized (i.e., location, date, and size), they can be configured as input for WFEIS simulations as custom compiled shapefiles in cases where they are not in existing spatial datasets. The most recent FCCS fuel layer (LANDFIRE (v2.2.0), [45]) includes the sole fuelbed described as a prescribed burn, which is on longleaf pine stands.

### Carbon mass emitted relative to carbon stock in trees

Forest inventory data can contribute to development of fire fuels models. However, the data and models used to estimate forest fire emissions are largely independent of the data or models used to estimate forest carbon stocks. The NIR reporting includes an implicit assumption of consistency among parts, but past NIRs have not directly addressed the magnitude of carbon emitted with forest fires relative to carbon mass in forests prior to fires. The informal analysis here (Fig. 4) suggests similar magnitudes of carbon mass. Actual fire emission at the inventory plot likely differs from the whole-fire mean just as aboveground tree carbon varies spatially and is not entirely consumed in many fires. Additionally, fuels consumed and contributing to emissions generally include dead wood and litter, which are not included in these summaries. Summaries suggest generally consistent and similar carbon levels despite only indirect links between forest inventory plots and the WFEIS system of models. These results suggest there is unlikely any serious disconnect between inventory and fire emissions, but better resolution would be useful. The uncertainty or high variability in comparing single value summaries of two highly variable quantities can be reduced somewhat by specifying relatively small burned areas of interest centered over permenant inventory plots measured before and after fires.

## Conclusion

The WFEIS system provides emissions estimates that conform to the reporting criteria – fire on forest land for CONUS plus Alaska over 1990 through 2022. The system is available on the web, and is compatible with the scope and reporting needs of the NIR. Future development may include additional burn sources, particularly to fill in missing smaller fires, which constitute a relatively minor contribution to total fire emissions. An additional, but possibly longer-term improvement goal, is to identify and include prescribed forest fires in the estimates.

### Electronic supplementary material

Below is the link to the electronic supplementary material.

Supplementary Material 1

## Data Availability

No datasets were generated or analysed during the current study.

## Abbreviations

WFEIS: Wildland Fire Emissions Inventory System
NIR: National Greenhouse Gas Inventory Report -- Inventory of U.S. Greenhouse Gas Emissions and Sinks: 1990–2022
CONUS: The 48 conterminous states of the United States
MTBS: Monitoring Trends in Burn Severity
MODIS: Moderate Resolution Imaging Spectroradiometer burned area mapping product
WFIGS: Wildland Fire Interagency Geospatial Service Interagency Fire Perimeters
FCCS: Fuel Characteristic Classification System
NAWFD: North American Wildland Fuels Database
