# Supplementary material for: Application of the wildland fire emissions inventory system to estimate fire emissions on forest lands of the United States
Source: Carbon Balance Manag. 2024 Aug 14;19:26. doi: 10.1186/s13021-024-00274-0 (PMC11325709; doi:10.1186/s13021-024-00274-0)
Supplement: Supplementary file 1 — Supplementary Material 1 [file 13021_2024_274_MOESM1_ESM.docx]

Additional file tables

Application of the Wildland Fire Emissions Inventory System to estimate fire emissions on forest lands of the United States

Smith, Billmire, French, and Domke

Abbreviations in Tables S1-S3

MTBS Monitoring Trends in Burn Severity

MODIS Moderate Resolution Imaging Spectroradiometer burned area mapping product

WFIGS Wildland Fire Interagency Geospatial Service Interagency Fire Perimeters

FCCS Fuel Characteristic Classification System

NAWFD North American Wildland Fuels Database

Table S1. Burn perimeter relative size and number characteristics example from the 2021 CONUS perimeters.

| Source | Minimum | Median | 99^th^ percentile | Number of 2021 perimeters |
| --- | --- | --- | --- | --- |
|  |  | Perimeter ha |  |  |
| MTBS | 202 | 714 | 46395 | 1003 |
| MODIS | 21 | 21 | 473 | 69051 |
| WFIGS | 0.2 | 2 | 10676 | 5158 |

Note: These are full burn perimeters, not the forest fires subset. The minimum MTBS size is set by MTBS; the minimum MODIS size is a single MODIS scene; and we truncated the WFIGS records to a minimum of 0.2 ha for this analysis (actual minimum is <0.0001 ha). The number of WFIGS perimeters reflects the truncated set.

Table S2. Annual WFEIS-based estimates of area of forest fires summarized by region with estimates for combinations of burn data by fuel source. Note cells are filled according to source data availability.

| Year | MTBS & FCCS | MTBS & NAWFD | MODIS & FCCS | MODIS & NAWFD | WFIGS & FCCS | WFIGS & NAWFD |
| --- | --- | --- | --- | --- | --- | --- |
|  |  |  | thousand ha |  |  |  |
|  |  |  |  |  |  |  |
| Alaska |  |  |  |  |  |  |
| 1990 | 529 | 518 | - | - | - | - |
| 1991 | 187 | 183 | - | - | - | - |
| 1992 | 16 | 15 | - | - | - | - |
| 1993 | 100 | 101 | - | - | - | - |
| 1994 | 49 | 50 | - | - | - | - |
| 1995 | 7 | 7 | - | - | - | - |
| 1996 | 66 | 66 | - | - | - | - |
| 1997 | 15 | 16 | - | - | - | - |
| 1998 | 38 | 39 | - | - | - | - |
| 1999 | 126 | 215 | - | - | - | - |
| 2000 | 147 | 148 | - | - | - | - |
| 2001 | 37 | 37 | 1 | 1 | - | - |
| 2002 | 459 | 457 | 321 | 319 | - | - |
| 2003 | 176 | 169 | 114 | 110 | - | - |
| 2004 | 1,208 | 1,209 | 1,506 | 1,490 | - | - |
| 2005 | 784 | 766 | 985 | 978 | - | - |
| 2006 | 20 | 20 | 19 | 19 | - | - |
| 2007 | 79 | 79 | 61 | 61 | - | - |
| 2008 | 21 | 21 | 10 | 10 | - | - |
| 2009 | 573 | 569 | 506 | 499 | - | - |
| 2010 | 57 | 72 | 45 | 51 | - | - |
| 2011 | 45 | 48 | 6 | 6 | - | - |
| 2012 | 4 | 6 | 20 | 22 | - | - |
| 2013 | 325 | 375 | 159 | 176 | - | - |
| 2014 | 2 | 2 | 2 | 5 | - | - |
| 2015 | 792 | 890 | 812 | 926 | - | - |
| 2016 | 97 | 22 | 69 | 14 | - | - |
| 2017 | 127 | 113 | 113 | 105 | - | - |
| 2018 | 112 | 108 | 66 | 66 | - | - |
| 2019 | 98 | 90 | 522 | 498 | - | - |
| 2020 | 10 | 8 | 7 | 6 | - | - |
| 2021 | - | - | 50 | 48 | 80 | 73 |
| 2022 | - | - | 417 | 388 | 836 | 769 |
|  |  |  |  |  |  |  |
| PacCoast |  |  |  |  |  |  |
| 1990 | 30 | 53 | - | - | - | - |
| 1991 | 9 | 11 | - | - | - | - |
| 1992 | 40 | 57 | - | - | - | - |
| 1993 | 1 | 10 | - | - | - | - |
| 1994 | 78 | 91 | - | - | - | - |
| 1995 | 5 | 8 | - | - | - | - |
| 1996 | 74 | 123 | - | - | - | - |
| 1997 | 9 | 20 | - | - | - | - |
| 1998 | 8 | 10 | - | - | - | - |
| 1999 | 94 | 125 | - | - | - | - |
| 2000 | 53 | 61 | - | - | - | - |
| 2001 | 69 | 77 | 76 | 85 | - | - |
| 2002 | 155 | 182 | 292 | 328 | - | - |
| 2003 | 123 | 142 | 123 | 143 | - | - |
| 2004 | 46 | 54 | 60 | 70 | - | - |
| 2005 | 34 | 45 | 29 | 40 | - | - |
| 2006 | 172 | 177 | 173 | 203 | - | - |
| 2007 | 100 | 128 | 135 | 195 | - | - |
| 2008 | 239 | 292 | 171 | 219 | - | - |
| 2009 | 50 | 85 | 49 | 85 | - | - |
| 2010 | 15 | 17 | 19 | 20 | - | - |
| 2011 | 28 | 32 | 25 | 29 | - | - |
| 2012 | 136 | 147 | 128 | 136 | - | - |
| 2013 | 157 | 176 | 131 | 150 | - | - |
| 2014 | 134 | 143 | 146 | 155 | - | - |
| 2015 | 401 | 477 | 375 | 453 | - | - |
| 2016 | 68 | 35 | 73 | 47 | - | - |
| 2017 | 242 | 253 | 327 | 360 | - | - |
| 2018 | 227 | 276 | 346 | 442 | - | - |
| 2019 | 36 | 37 | 62 | 62 | - | - |
| 2020 | 238 | 236 | 1,117 | 1,120 | - | - |
| 2021 | 113 | 109 | 1,101 | 1,003 | 1,249 | 1,148 |
| 2022 | - | - | 199 | 184 | 233 | 222 |
|  |  |  |  |  |  |  |
| RockyMtn |  |  |  |  |  |  |
| 1990 | 19 | 19 | - | - | - | - |
| 1991 | 23 | 18 | - | - | - | - |
| 1992 | 26 | 24 | - | - | - | - |
| 1993 | 38 | 39 | - | - | - | - |
| 1994 | 176 | 161 | - | - | - | - |
| 1995 | 37 | 37 | - | - | - | - |
| 1996 | 97 | 90 | - | - | - | - |
| 1997 | 15 | 15 | - | - | - | - |
| 1998 | 36 | 34 | - | - | - | - |
| 1999 | 45 | 42 | - | - | - | - |
| 2000 | 510 | 464 | - | - | - | - |
| 2001 | 117 | 105 | 80 | 70 | - | - |
| 2002 | 212 | 208 | 361 | 367 | - | - |
| 2003 | 346 | 336 | 319 | 304 | - | - |
| 2004 | 67 | 66 | 62 | 59 | - | - |
| 2005 | 108 | 109 | 122 | 111 | - | - |
| 2006 | 284 | 253 | 270 | 250 | - | - |
| 2007 | 424 | 399 | 535 | 483 | - | - |
| 2008 | 79 | 70 | 95 | 82 | - | - |
| 2009 | 77 | 77 | 86 | 78 | - | - |
| 2010 | 39 | 41 | 37 | 35 | - | - |
| 2011 | 154 | 138 | 323 | 311 | - | - |
| 2012 | 443 | 420 | 490 | 477 | - | - |
| 2013 | 155 | 147 | 231 | 220 | - | - |
| 2014 | 103 | 96 | 78 | 72 | - | - |
| 2015 | 205 | 192 | 200 | 190 | - | - |
| 2016 | 282 | 111 | 255 | 98 | - | - |
| 2017 | 417 | 340 | 400 | 328 | - | - |
| 2018 | 302 | 240 | 362 | 261 | - | - |
| 2019 | 74 | 54 | 87 | 64 | - | - |
| 2020 | 165 | 145 | 379 | 315 | - | - |
| 2021 | 152 | 144 | 343 | 290 | 383 | 353 |
| 2022 | - | - | 345 | 310 | 412 | 378 |
|  |  |  |  |  |  |  |
| North |  |  |  |  |  |  |
| 1990 | 22 | 20 | - | - | - | - |
| 1991 | 159 | 162 | - | - | - | - |
| 1992 | 13 | 7 | - | - | - | - |
| 1993 | 2 | 1 | - | - | - | - |
| 1994 | 24 | 23 | - | - | - | - |
| 1995 | 22 | 14 | - | - | - | - |
| 1996 | 9 | 7 | - | - | - | - |
| 1997 | 4 | 2 | - | - | - | - |
| 1998 | 9 | 8 | - | - | - | - |
| 1999 | 40 | 35 | - | - | - | - |
| 2000 | 18 | 17 | - | - | - | - |
| 2001 | 28 | 27 | 17 | 18 | - | - |
| 2002 | 8 | 6 | 9 | 7 | - | - |
| 2003 | 17 | 15 | 34 | 33 | - | - |
| 2004 | 11 | 9 | 17 | 16 | - | - |
| 2005 | 21 | 20 | 17 | 16 | - | - |
| 2006 | 28 | 25 | 26 | 25 | - | - |
| 2007 | 25 | 20 | 30 | 25 | - | - |
| 2008 | 5 | 5 | 8 | 7 | - | - |
| 2009 | 9 | 9 | 30 | 29 | - | - |
| 2010 | 22 | 21 | 46 | 45 | - | - |
| 2011 | 25 | 25 | 56 | 56 | - | - |
| 2012 | 13 | 13 | 47 | 46 | - | - |
| 2013 | 14 | 14 | 10 | 10 | - | - |
| 2014 | 9 | 8 | 11 | 11 | - | - |
| 2015 | 19 | 18 | 15 | 14 | - | - |
| 2016 | 15 | 10 | 21 | 14 | - | - |
| 2017 | 9 | 7 | 13 | 8 | - | - |
| 2018 | 15 | 9 | 25 | 20 | - | - |
| 2019 | 18 | 12 | 27 | 16 | - | - |
| 2020 | 10 | 6 | 15 | 12 | - | - |
| 2021 | 33 | 27 | 33 | 34 | 30 | 27 |
| 2022 | - | - | 17 | 13 | 10 | 8 |
|  |  |  |  |  |  |  |
| South |  |  |  |  |  |  |
| 1990 | 46 | 19 | - | - | - | - |
| 1991 | 102 | 53 | - | - | - | - |
| 1992 | 65 | 14 | - | - | - | - |
| 1993 | 36 | 12 | - | - | - | - |
| 1994 | 70 | 32 | - | - | - | - |
| 1995 | 47 | 29 | - | - | - | - |
| 1996 | 86 | 18 | - | - | - | - |
| 1997 | 35 | 11 | - | - | - | - |
| 1998 | 167 | 45 | - | - | - | - |
| 1999 | 151 | 99 | - | - | - | - |
| 2000 | 153 | 84 | - | - | - | - |
| 2001 | 150 | 96 | 118 | 70 | - | - |
| 2002 | 90 | 65 | 140 | 77 | - | - |
| 2003 | 83 | 58 | 240 | 114 | - | - |
| 2004 | 173 | 65 | 325 | 182 | - | - |
| 2005 | 224 | 99 | 271 | 134 | - | - |
| 2006 | 299 | 155 | 312 | 140 | - | - |
| 2007 | 514 | 323 | 443 | 271 | - | - |
| 2008 | 349 | 123 | 328 | 178 | - | - |
| 2009 | 320 | 110 | 335 | 135 | - | - |
| 2010 | 400 | 184 | 244 | 124 | - | - |
| 2011 | 468 | 186 | 597 | 295 | - | - |
| 2012 | 160 | 57 | 199 | 83 | - | - |
| 2013 | 237 | 126 | 280 | 139 | - | - |
| 2014 | 291 | 145 | 268 | 121 | - | - |
| 2015 | 188 | 86 | 240 | 118 | - | - |
| 2016 | 372 | 188 | 356 | 138 | - | - |
| 2017 | 326 | 138 | 392 | 138 | - | - |
| 2018 | 309 | 99 | 330 | 83 | - | - |
| 2019 | 150 | 53 | 277 | 74 | - | - |
| 2020 | 141 | 48 | 212 | 51 | - | - |
| 2021 | 337 | 137 | 314 | 100 | 103 | 33 |
| 2022 | - | - | 344 | 117 | 88 | 34 |

Table S3. Annual WFEIS-based estimates of CO_2_ emitted with forest fires summarized by region with estimates for combinations of burn data by fuel source. Note cells are filled according to source data availability.

| Year | MTBS & FCCS | MTBS & NAWFD | MODIS & FCCS | MODIS & NAWFD | WFIGS & FCCS | WFIGS & NAWFD |
| --- | --- | --- | --- | --- | --- | --- |
|  |  |  | Tg CO_2_ |  |  |  |
|  |  |  |  |  |  |  |
| Alaska |  |  |  |  |  |  |
| 1990 | 92.5 | 72.3 |  |  |  |  |
| 1991 | 34.2 | 24.5 |  |  |  |  |
| 1992 | 2.3 | 2.0 |  |  |  |  |
| 1993 | 20.6 | 13.1 |  |  |  |  |
| 1994 | 12.0 | 6.2 |  |  |  |  |
| 1995 | 1.2 | 0.9 |  |  |  |  |
| 1996 | 12.8 | 8.4 |  |  |  |  |
| 1997 | 3.2 | 2.1 |  |  |  |  |
| 1998 | 12.1 | 4.9 |  |  |  |  |
| 1999 | 35.3 | 16.3 |  |  |  |  |
| 2000 | 38.3 | 18.1 |  |  |  |  |
| 2001 | 9.8 | 4.7 | 0.2 | 0.2 |  |  |
| 2002 | 118.6 | 59.3 | 82.0 | 41.2 |  |  |
| 2003 | 33.7 | 21.8 | 21.5 | 13.7 |  |  |
| 2004 | 288.4 | 166.1 | 337.6 | 216.7 |  |  |
| 2005 | 173.9 | 100.5 | 229.9 | 132.8 |  |  |
| 2006 | 4.9 | 3.0 | 3.9 | 2.8 |  |  |
| 2007 | 19.0 | 11.4 | 13.7 | 8.4 |  |  |
| 2008 | 5.1 | 2.5 | 2.2 | 1.2 |  |  |
| 2009 | 139.4 | 74.4 | 119.6 | 68.7 |  |  |
| 2010 | 14.8 | 9.2 | 9.3 | 6.7 |  |  |
| 2011 | 10.3 | 6.3 | 1.3 | 0.8 |  |  |
| 2012 | 0.7 | 0.8 | 2.9 | 2.8 |  |  |
| 2013 | 85.6 | 50.4 | 40.9 | 23.0 |  |  |
| 2014 | 0.3 | 0.3 | 0.6 | 0.6 |  |  |
| 2015 | 199.2 | 120.9 | 204.2 | 123.2 |  |  |
| 2016 | 6.2 | 3.2 | 4.2 | 2.0 |  |  |
| 2017 | 24.2 | 17.9 | 20.0 | 17.6 |  |  |
| 2018 | 19.0 | 12.5 | 11.9 | 7.7 |  |  |
| 2019 | 19.4 | 12.2 | 86.5 | 72.5 |  |  |
| 2020 | 1.0 | 1.1 | 0.6 | 0.9 |  |  |
| 2021 |  |  | 8.9 | 6.1 | 14.9 | 9.2 |
| 2022 |  |  | 72.0 | 53.1 | 145.9 | 103.6 |
|  |  |  |  |  |  |  |
| PacCoast |  |  |  |  |  |  |
| 1990 | 3.0 | 7.6 |  |  |  |  |
| 1991 | 1.1 | 1.9 |  |  |  |  |
| 1992 | 4.2 | 8.0 |  |  |  |  |
| 1993 | 0.2 | 1.6 |  |  |  |  |
| 1994 | 6.5 | 13.6 |  |  |  |  |
| 1995 | 0.7 | 1.6 |  |  |  |  |
| 1996 | 8.3 | 18.6 |  |  |  |  |
| 1997 | 1.1 | 3.2 |  |  |  |  |
| 1998 | 0.6 | 1.4 |  |  |  |  |
| 1999 | 8.7 | 20.3 |  |  |  |  |
| 2000 | 4.6 | 8.4 |  |  |  |  |
| 2001 | 6.7 | 11.5 | 7.0 | 12.3 |  |  |
| 2002 | 18.5 | 29.0 | 36.7 | 55.6 |  |  |
| 2003 | 13.4 | 23.9 | 13.8 | 23.6 |  |  |
| 2004 | 3.8 | 7.9 | 5.9 | 10.6 |  |  |
| 2005 | 3.4 | 7.1 | 3.0 | 6.1 |  |  |
| 2006 | 15.6 | 27.1 | 16.9 | 31.0 |  |  |
| 2007 | 9.6 | 17.9 | 12.7 | 27.1 |  |  |
| 2008 | 26.4 | 54.0 | 19.2 | 39.4 |  |  |
| 2009 | 6.6 | 15.0 | 6.5 | 14.4 |  |  |
| 2010 | 1.6 | 2.7 | 2.3 | 3.4 |  |  |
| 2011 | 3.3 | 5.6 | 2.9 | 4.8 |  |  |
| 2012 | 13.5 | 23.0 | 13.5 | 21.3 |  |  |
| 2013 | 19.2 | 30.3 | 15.2 | 24.9 |  |  |
| 2014 | 11.5 | 22.5 | 12.2 | 23.0 |  |  |
| 2015 | 33.0 | 75.4 | 29.7 | 68.6 |  |  |
| 2016 | 4.3 | 4.8 | 6.6 | 7.4 |  |  |
| 2017 | 31.1 | 49.4 | 35.1 | 66.9 |  |  |
| 2018 | 19.1 | 43.3 | 26.7 | 70.1 |  |  |
| 2019 | 2.5 | 4.6 | 5.0 | 8.7 |  |  |
| 2020 | 23.2 | 40.3 | 125.2 | 208.8 |  |  |
| 2021 | 10.5 | 16.7 | 114.4 | 175.1 | 132.5 | 202.6 |
| 2022 |  |  | 24.8 | 33.0 | 30.0 | 39.9 |
|  |  |  |  |  |  |  |
| RockyMtn |  |  |  |  |  |  |
| 1990 | 1.2 | 2.0 |  |  |  |  |
| 1991 | 2.1 | 2.3 |  |  |  |  |
| 1992 | 1.7 | 2.7 |  |  |  |  |
| 1993 | 2.0 | 3.6 |  |  |  |  |
| 1994 | 14.3 | 21.6 |  |  |  |  |
| 1995 | 2.0 | 3.7 |  |  |  |  |
| 1996 | 5.9 | 9.5 |  |  |  |  |
| 1997 | 0.8 | 1.4 |  |  |  |  |
| 1998 | 2.7 | 3.7 |  |  |  |  |
| 1999 | 2.6 | 3.9 |  |  |  |  |
| 2000 | 40.4 | 64.4 |  |  |  |  |
| 2001 | 10.0 | 12.5 | 7.7 | 8.8 |  |  |
| 2002 | 15.5 | 23.3 | 22.5 | 38.3 |  |  |
| 2003 | 31.1 | 42.3 | 28.6 | 38.3 |  |  |
| 2004 | 4.3 | 7.5 | 3.9 | 6.3 |  |  |
| 2005 | 7.6 | 12.4 | 9.8 | 13.3 |  |  |
| 2006 | 21.2 | 30.3 | 20.5 | 29.3 |  |  |
| 2007 | 35.3 | 53.6 | 47.2 | 66.8 |  |  |
| 2008 | 6.3 | 8.2 | 7.7 | 9.4 |  |  |
| 2009 | 4.5 | 7.4 | 5.3 | 8.0 |  |  |
| 2010 | 2.4 | 4.1 | 2.4 | 3.5 |  |  |
| 2011 | 12.1 | 15.9 | 20.9 | 34.4 |  |  |
| 2012 | 37.8 | 51.6 | 39.4 | 54.5 |  |  |
| 2013 | 13.4 | 19.2 | 20.5 | 29.2 |  |  |
| 2014 | 5.9 | 9.4 | 5.2 | 7.3 |  |  |
| 2015 | 19.0 | 23.7 | 20.0 | 25.7 |  |  |
| 2016 | 13.6 | 11.2 | 15.2 | 10.9 |  |  |
| 2017 | 33.2 | 39.6 | 34.9 | 39.6 |  |  |
| 2018 | 21.7 | 25.5 | 25.7 | 28.7 |  |  |
| 2019 | 3.7 | 4.8 | 5.7 | 6.3 |  |  |
| 2020 | 8.1 | 14.5 | 19.3 | 32.1 |  |  |
| 2021 | 11.1 | 16.2 | 26.5 | 33.4 | 28.6 | 41.4 |
| 2022 |  |  | 24.7 | 33.5 | 26.6 | 39.3 |
|  |  |  |  |  |  |  |
| North |  |  |  |  |  |  |
| 1990 | 4.5 | 1.8 |  |  |  |  |
| 1991 | 14.6 | 17.4 |  |  |  |  |
| 1992 | 0.9 | 0.6 |  |  |  |  |
| 1993 | 0.1 | 0.1 |  |  |  |  |
| 1994 | 1.8 | 2.4 |  |  |  |  |
| 1995 | 1.7 | 1.5 |  |  |  |  |
| 1996 | 0.8 | 0.7 |  |  |  |  |
| 1997 | 0.2 | 0.2 |  |  |  |  |
| 1998 | 1.5 | 0.8 |  |  |  |  |
| 1999 | 3.6 | 3.8 |  |  |  |  |
| 2000 | 1.8 | 1.6 |  |  |  |  |
| 2001 | 2.4 | 2.9 | 1.5 | 1.8 |  |  |
| 2002 | 0.7 | 0.6 | 0.5 | 0.7 |  |  |
| 2003 | 3.0 | 1.5 | 2.6 | 3.4 |  |  |
| 2004 | 1.2 | 0.9 | 1.1 | 1.6 |  |  |
| 2005 | 2.5 | 2.1 | 1.3 | 1.6 |  |  |
| 2006 | 3.7 | 2.7 | 3.5 | 2.7 |  |  |
| 2007 | 5.4 | 1.9 | 5.1 | 2.5 |  |  |
| 2008 | 0.4 | 0.5 | 0.5 | 0.7 |  |  |
| 2009 | 0.7 | 0.9 | 2.2 | 2.8 |  |  |
| 2010 | 3.5 | 2.1 | 5.0 | 4.5 |  |  |
| 2011 | 8.7 | 2.6 | 17.1 | 5.9 |  |  |
| 2012 | 2.0 | 1.3 | 12.2 | 4.5 |  |  |
| 2013 | 1.2 | 1.4 | 0.7 | 0.9 |  |  |
| 2014 | 0.6 | 0.8 | 0.8 | 1.0 |  |  |
| 2015 | 2.3 | 1.8 | 1.2 | 1.3 |  |  |
| 2016 | 1.0 | 1.0 | 1.2 | 1.4 |  |  |
| 2017 | 0.5 | 0.6 | 0.8 | 0.8 |  |  |
| 2018 | 1.8 | 0.8 | 1.7 | 1.8 |  |  |
| 2019 | 1.0 | 1.2 | 1.6 | 1.6 |  |  |
| 2020 | 0.4 | 0.6 | 0.8 | 1.1 |  |  |
| 2021 | 1.8 | 2.6 | 4.2 | 3.3 | 4.0 | 2.6 |
| 2022 |  |  | 0.9 | 1.3 | 0.6 | 0.8 |
|  |  |  |  |  |  |  |
| South |  |  |  |  |  |  |
| 1990 | 1.9 | 1.7 |  |  |  |  |
| 1991 | 6.0 | 5.4 |  |  |  |  |
| 1992 | 2.4 | 1.3 |  |  |  |  |
| 1993 | 1.3 | 1.0 |  |  |  |  |
| 1994 | 3.4 | 3.1 |  |  |  |  |
| 1995 | 2.6 | 2.7 |  |  |  |  |
| 1996 | 3.5 | 1.4 |  |  |  |  |
| 1997 | 1.3 | 0.8 |  |  |  |  |
| 1998 | 5.9 | 3.9 |  |  |  |  |
| 1999 | 9.1 | 10.0 |  |  |  |  |
| 2000 | 8.4 | 8.4 |  |  |  |  |
| 2001 | 9.7 | 9.6 | 6.4 | 6.5 |  |  |
| 2002 | 5.0 | 6.3 | 6.6 | 6.9 |  |  |
| 2003 | 4.5 | 5.6 | 11.5 | 9.9 |  |  |
| 2004 | 7.4 | 5.7 | 15.7 | 14.8 |  |  |
| 2005 | 10.9 | 7.6 | 13.3 | 11.6 |  |  |
| 2006 | 14.4 | 13.3 | 14.2 | 11.2 |  |  |
| 2007 | 25.7 | 31.5 | 21.5 | 24.6 |  |  |
| 2008 | 13.7 | 8.7 | 16.2 | 14.5 |  |  |
| 2009 | 14.7 | 8.4 | 15.2 | 10.9 |  |  |
| 2010 | 17.8 | 14.3 | 11.7 | 10.6 |  |  |
| 2011 | 23.0 | 16.5 | 29.4 | 25.2 |  |  |
| 2012 | 7.2 | 4.8 | 9.0 | 7.0 |  |  |
| 2013 | 11.1 | 10.1 | 12.7 | 10.7 |  |  |
| 2014 | 13.3 | 11.3 | 12.2 | 9.8 |  |  |
| 2015 | 8.4 | 6.5 | 11.4 | 9.4 |  |  |
| 2016 | 17.1 | 17.8 | 16.5 | 12.4 |  |  |
| 2017 | 15.4 | 11.7 | 18.8 | 11.5 |  |  |
| 2018 | 13.5 | 8.0 | 14.3 | 6.3 |  |  |
| 2019 | 6.9 | 4.5 | 12.4 | 5.7 |  |  |
| 2020 | 5.1 | 4.1 | 7.9 | 4.1 |  |  |
| 2021 | 14.6 | 12.0 | 13.0 | 8.6 | 6.0 | 2.8 |
| 2022 |  |  | 14.6 | 10.1 | 4.1 | 2.9 |
